# TCR repertoire and transcriptional signatures of circulating tumour‐associated T cells facilitate effective non‐invasive cancer detection

**DOI:** 10.1002/ctm2.853

**Published:** 2022-09-22

**Authors:** Fansen Ji, Lin Chen, Zhizhuo Chen, Bin Luo, Yongwang Wang, Xun Lan

**Affiliations:** ^1^ Tsinghua‐Peking Center for Life Sciences MOE Key Laboratory of Tsinghua University Beijing China; ^2^ School of Medicine Tsinghua University Beijing China; ^3^ General Surgery Department Beijing Tsinghua Changgung Hospital School of Clinical Medicine Tsinghua University Beijing China; ^4^ School of Life Science Tsinghua University Beijing China; ^5^ Department of Anesthesiology Affiliated Hospital of Guilin Medical University Guilin China

1

The concept of precision medicine in oncology has dramatically transformed the clinical application of tumour screening, which makes the malignancies more curable if diagnosed early. Traditional serological tumour biomarkers like α‐fetoprotein, prostate‐specific antigens, carcinoembryonic antigens, CA19–9 and CA125 have been widely investigated in clinic, but the specificity has not reached a satisfactory stage for population level.^1^, ^2^, ^3^ Novel technologies utilizing tumour‐derived signals from blood non‐invasively provide a new tumour diagnostic strategy called liquid biopsy over the past decades.^3^, ^4^, ^5^ Several peripheral biomarkers such as cell‐free DNA (cfDNA)^6^, ^7^, ^8^ especially circulating tumour DNA (ctDNA),^9^ circulating tumour cells (CTCs),^10^, ^11^ circulating micro‐RNAs,^12^, ^13^ tumour‐derived exosomes^14^ and cancer cell metabolites^15^ achieved great progress and showed huge prospects in tumour screening. However, these methods are all derived from the modality of tumour and often need predefined panels or biomarkers for diagnosis, which may be non‐specific and subjective due to the heterogeneous properties of tumour. The feasibility of using tumour‐associated T cell response involved in tumour initiation and development, as a supplementary diagnosis choice has not been explored widely.^16^, ^17^

Until recently, tumour‐infiltrated T lymphocytes (TILs) were considered to be beneficial tumour‐specific T cells.^18^ But due to the complex interaction of different immune components mediated by chemokines or cytokines within the tumour microenvironment (TME), the majority of passively expanding TILs cannot recognize tumour‐specific antigens (TSAs) and are thus believed to be bystander T cells.^19^, ^20^, ^21^ These bystander T cells may dilute tumour‐specific signals and make the identification of TSAs‐specific T cells challenging.^22^, ^23^, ^24^ Programmed cell death protein 1 (PD‐1) is suggested to be a biomarker for tumour‐specific CD8^+^ T cells both in TILs and in peripheral blood mononuclear cells (PBMCs),^22^, ^25^, ^26^, ^27^ but the efficacy needs to be further validated in practical applications.^21^, ^28^, ^29^ Tracking the general immunophenotype of T lymphocytes when they encounter antigens and enrichment of tumour‐associated T cells over a pool of irrelevant signals during tumour development reflects the overall immune status of patients and offers opportunities for cancer prevention and therapy.^30^

Next generation sequencing (NGS)‐based T cell receptor (TCR) repertoire quantification has provided methods for TSA recognition and now is extensively used in the identification of tumour‐reactive T lymphocytes.^19^, ^31^, ^32^, ^33^, ^34^ The past few years have witnessed a series of studies utilizing T or B‐cell repertoire to pinpoint disease‐associated signatures, and evidences have demonstrated the diagnostic potential of TCR repertoire in autoimmune diseases,^35^ infectious diseases^36^, ^37^ and even cancer.^38^, ^39^, ^40^, ^41^, ^42^ Sustained neoantigen stimulation during tumour cell development impels TCR to shift towards a tumour‐specific distribution and to exhibit different amino acids motifs than those in healthy cells.^38^, ^43^

Under physiological conditions, naïve T cells maturing in the thymus will flow through peripheral blood or lymphatic vessels and migrate through high endothelial venules into secondary lymphoid organs where they encounter potential tumour antigens.^44^, ^45^ T cell trafficking and circulation theoretically enable these tumour‐specific T cells to be detectable both in tumour sites and peripheral blood. T lymphocytes circulating among PBMCs that paired with TILs residing in tumour tissues have been suggested to be highly correlated with T cell‐induced cytotoxicity and to indicate enrichment of tumour reactive signals.^22^, ^46^, ^47^, ^48^, ^49^, ^50^, ^51^ Elucidating the connection between anti‐tumour T cells in the periphery and those in the TME^22^, ^25^, ^44^, ^45^, ^52^ may provide clues to design novel approaches for non‐invasive tumour screening. Assessing overlapping TCRs between PBMCs and TILs and considering them tumour‐specific predictors will not only help us comprehensively study T cell circulation and migration but will alleviate the deficiencies caused by using the TIL population only, which is enriched with bystander T cells.

In this study, we defined a group of circulating T lymphocytes in PBMCs that shared TCRs with TILs as tumour‐associated T cells (TATs). Using the CDR3 sequences of TATs with those in healthy TCRs as input data, we trained a binary model to distinguish TATs from healthy clones. Applying this model on several independent clinical datasets, we acquired the number of TAT sequences in PBMCs for each individual. We then designed a TCR repertoire risk score (TRRS) as the number of TATs our model predicted in the PBMCs divided by the number of detected healthy TCRs from healthy individuals. We demonstrated that the TRRS separated tumour patients from healthy donors effectively. Next, we characterized the transcriptional signatures of TATs in the PBMC populations using multiple single cell RNA sequencing (scRNA‐seq) coupled with TCR sequencing datasets and found that T cell activation pathway was significantly up‐regulated in TATs. Combining the TCR repertoire and transcriptional signatures of TATs,^53^ we developed an integrated framework for non‐invasive tumour screening using only PBMC samples. Furthermore, we performed bulk TCR and RNA sequencing of PBMC samples from 11 tumour patients and six healthy donors and validated the performance of this tumour screening strategy with these data and another independent cohort. Our study proves the principle of using TATs as an alternative non‐invasive tumour screening biomarker and broadens the liquid biopsy application from the view of the immune landscape.

T cells with identical TCR sequences are thought to be derived from a single naïve T cell, which migrates and circulates among different tissue types and may undergo a functional state transition upon antigen stimulation.^54^ Based on the TCR‐sharing relationship of TILs and PBMCs, we first divided TCR clonotypes in PBMCs and TILs into four different compartments (Figure 1A). TCR clonotypes of PBMCs that are identical to those of TILs are called *PBMCs_Shared* compartment, while clonotypes of TILs that are identical to those of PBMCs are called *TILs_Shared* compartment. These two compartments have the same TCR clonotypes, but their tissue sources distinguish them. Due to different tissue environment, the frequency of each clonotype and the degree of clonality between the two compartments may differ. Therefore, we specifically named the T cells among PBMCs that share TCRs with TILs as circulating Tumor Associated T cells (cTATs). In contrast, the TCR clonotypes that are unique to TILs are in the *TILs_only* compartment, and the TCR clonotypes observed only in PBMCs are in the *PBMCs_Only* compartment. We believe that the *PBMCs_Only* compartment most likely represents naïve, effector or memory T cells in periphery that are not related to the tumour immune response. T cells in the *TILs_Only* compartment may largely represent tissue‐resident T cells, which are not in the set of T lymphocytes prevalent in circulation. It should be noted that due to the technical limitation of TCR repertoire sequencing, both TILs_Only and PBMCs_Only compartments actually contain a proportion of overlapped clonotypes that cannot be detected sensitively at present.

**FIGURE 1:**
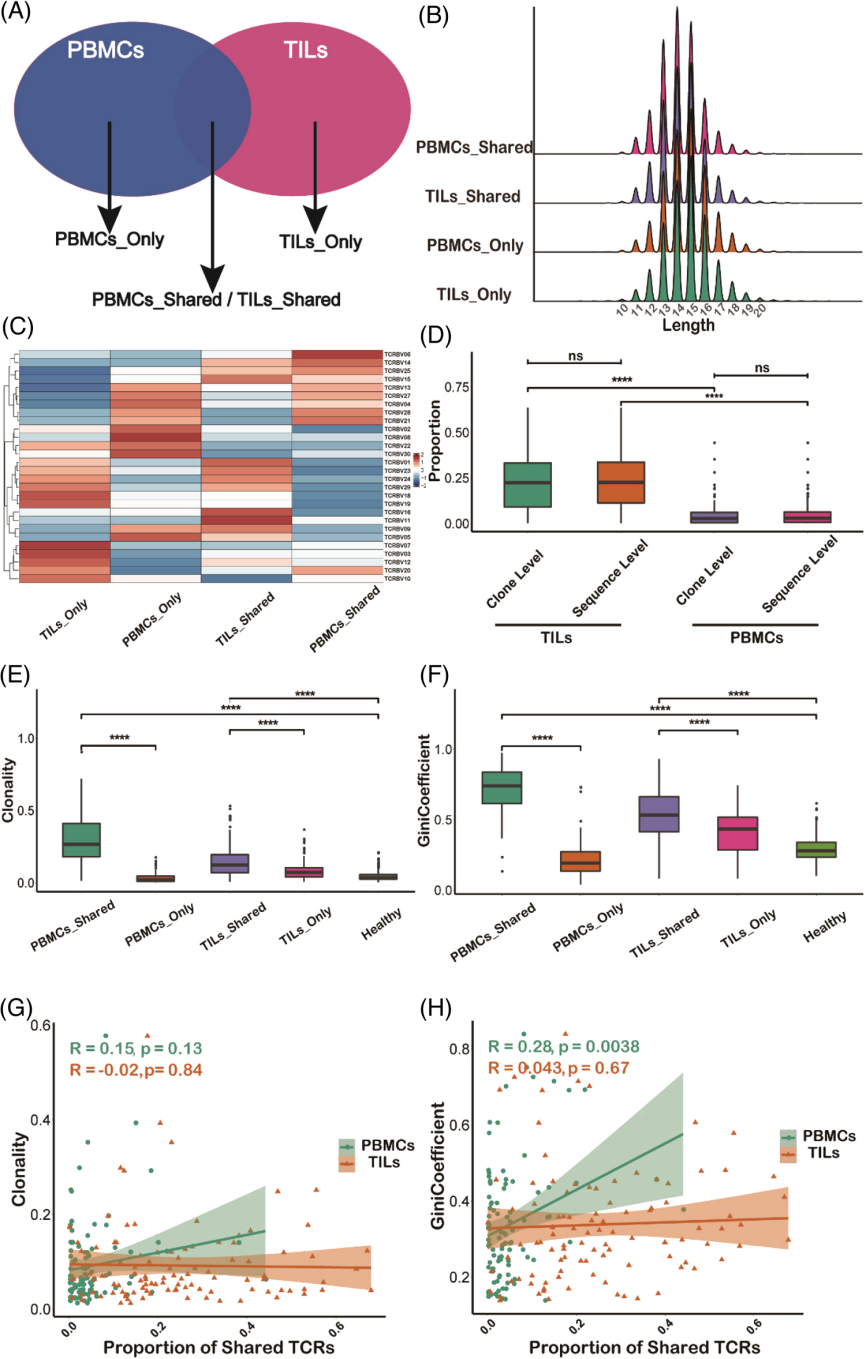
T cell receptor repertoire (TCR) sharing relationship between the tumour microenvironment, and the periphery defines four different compartments. (A) Schematic overview of TCR sharing relationship between PBMCs and tumour‐infiltrated T lymphocytes (TILs) and the definition of the four TCR compartments. (B) Length distribution of the CDR3 beta chains within the four different TCR compartments. (C) V gene usage of the CDR3 beta chains in different TCR compartments. (D) Proportion of shared TCRs among different tissue types (TILs or PBMCs) at both sequence level and clone level. (E–F) The indices of clonality and Gini coefficient among different TCR compartments and that from healthy donor PBMCs samples. (G–H) Correlation between the overall clonality/Gini coefficient and the proportion of shared TCRs in PBMCs and TILs

This framework allowed us to use TCR sequences as molecular barcodes to track and analyse the function of TATs among TILs and PBMCs. We collected a series of TCR CDR3 β chain sequencing data of paired PBMCs and tumour tissues from the same patient (Table S1) and assigned each T cell clonotype to one of the four compartments using the aforementioned definitions. After removing non‐functional TCRs, we found that, among the different compartments, most (91%) of the TCR CDR3 sequences were 12∼17 amino acids in length (Figure 1B). In addition, the CDR3 sequence length distribution in the TILs resembles that in the PBMCs, indicating that there is no CDR3 length difference between these cells in the two different tissue types.

Next, because TCR beta chain variable (TCRBV) genes contributed the most diversity to CDR3 sequences, we analysed the TCRBV gene usage in the different compartments. We found that TRBV genes, such as TRBV06/14/15/20/25, are expressed more frequently in the shared compartments than in the non‐shared compartments^43^ (Figure 1C), which suggests that the antigen specificities of TCRs may differ between these compartments. In the following analysis, subsets with CDR3 sequences of 12∼17 amino acids were analysed, and TCRs with excessively long or short CDR3 regions were removed.

To estimate the degree of TCR sequence overlap in PBMCs and TILs, we calculated the relative proportion of TCRs shared by both TILs and PBMCs in each sample. We found that the proportion of TCRs in the *TILs_Shared* compartment (approximately 22.56%, 95% confidence interval (CI): 12.82%–37.51%) was significantly higher (*p *< 1e^−4^) than the proportion in the cTATs of the PBMC population (approximately 3.08%, 95% CI: .64%–5.15%, Figure 1D), which indicated that TILs show higher shared TCR enrichment than PBMCs possibly due to the close interaction of T lymphocytes with TSAs in the TME. This result is consistent both at the TCR clone and TCR sequence level (Figure 1D). The same analysis based on single‐cell TCR sequencing (TCR‐seq) data showed no significant differences (*p *> .05) in the proportion of shared TCRs at either the clone or sequence level, possibly due to the limited number of cells captured in single cell TCR‐seq datasets, and many shared clones might be labelled as not shared (Figure S1A).

TCR sequences with a high degree of similarity and clonal expansion are more likely to recognize TSAs effectively. We found the indices of clonality and Gini coefficient in the *PBMCs_Shared* compartment were higher (*p *< 1e^−4^) than those in *PBMCs_Only* compartment (Figure 1E,F). The same trend was also observed in the PBMC population when single cell TCR‐seq data were analysed (Figure S1B). These results indicate that TATs are more likely to undergo clonal expansion and to represent functional tumour‐reactive T cells. Adding a healthy donor cohort PBMC dataset^36^ as the control (see Methods), we found that the clonality and Gini coefficient of the healthy samples were lower (*p *< 1e^−3^) than those of the shared compartments and higher than those of the tissue‐only compartments (Figure 1E,F), possibly due to the baseline immune activity that developed against common antigens in the surrounding environments, such as influenza virus or human cytomegalovirus (HCMV). Our results suggest T cell clones in the shared compartment are more likely to be tumour reactive and are different from those induced by non‐tumour antigens commonly present in healthy individuals.

Moreover, we found that the proportion of TATs in blood, which is observable only when tumour tissue is sequenced, was highly correlated with the overall T cell clonality and Gini coefficient of PBMCs, which were obtained non‐invasively; however, such correlation was not observed in TILs (Figure 1G,H). These results highlight the potential of using the T cell clonality and Gini coefficient of PBMCs as indicators of cancer development. TATs among PBMCs are more likely to reflect the clonal expansion of T lymphocytes in periphery, and a higher degree of shared TCR clones among PBMCs may indicate that more neoantigen‐specific T cells pre‐exist in the PBMCs.

It has been reported that a greater degree of PBMC‐TIL TCR repertoire overlap indicates an improved immune response and is associated with better clinical outcome of immunotherapy.^42^, ^47^, ^49^, ^55^ We believe that this compartment largely represents tumour reactive T cells and may serve as a biomarker to distinguish blood samples of cancer patients from those of healthy individuals. In this study, we sought to build a deep learning binary classifier to predict tumour‐reactive TCR sequences. To construct a training dataset for the model, we first downloaded a publicly available TCR sequencing data obtained from PBMC samples of healthy individuals^36^ as the control dataset and only used data from HCMV‐negative individuals to exclude potential tumour‐irrelevant immune signals. Two datasets of healthy cohorts were included in the analysis, and we named these sets Healthy351 and Healthy69 according to the number of samples after filtering. Since the Healthy351 included more healthy donors and TCRs (more than 30 million), we considered the TCRs in this cohort to be a healthy TCR pool and used these data to identify the TCR sequences that overlapped with those in the PBMCs from cancer patients. Then, we extracted TAT TCRs in PBMC samples from TIL‐PBMC‐paired TCR sequencing datasets described above and filtered TCRs that were also detected in Healthy351.

We labelled the remaining TAT TCRs as positive samples and the TCRs in Healthy351 as negative samples. The schematic workflow and experimental design are summarized in Figure S2A. Deep convolutional neural networks (CNNs) generally performed better in TCR pattern recognition studies ^56^, ^57^, ^58^; therefore, we encoded the CDR3 beta chain using the one‐hot encoding method and built a three‐layer CNN to distinguish the TCRs of TATs from those of healthy individuals. The output of the CNN is the probability of each input TCR sequence being the TCR of a TAT. Next, we generated a TRRS for each PBMC sample summarizing the number of TATs our model had predicted in PBMC relative to the number of healthy TCRs that had been detected in the Healthy351 dataset. We evaluated the performance of the TRRS for non‐invasive cancer detection with several independent PBMC datasets obtained from cancer patients using Healthy69 as negative samples. The detailed illustration of model construction is presented in the Methods.

We first selected the same number of negative TCRs as that of TATs and used five‐fold cross validation to test the generalization ability of our CNN model. Considering the heterogeneity of cancer patients, the data were split at the patient level rather than at the TCR sequence level to ensure that the model did not learn sample‐specific confounding effects. Both the receiver operating characteristic curve (ROC) curve and precision‐recall curve (PRC) (Figure 2A,B) showed the model performed modestly well in differentiating TCRs of TATs from TCRs of healthy samples (ROC: .699–.706, PRC: .446–.787) and are not influenced by human leukocyte antigen (HLA) haplotypes (Methods, Figure S2B,C). Because we randomly split patients into the training and test dataset and high variation in the number of TATs exists in different patients, the PRC shows high variability across the different iterations of random splits. The final model was trained and validated using the entire data, and as the number of training epochs increased to about 60, the loss and accuracy had reached a plateau (Figure 2C). Further inspection of the prediction probability distribution of different CDR3 length indicated a significant difference between the TCRs of TATs and those of healthy samples (Figure 2D).

**FIGURE 2:**
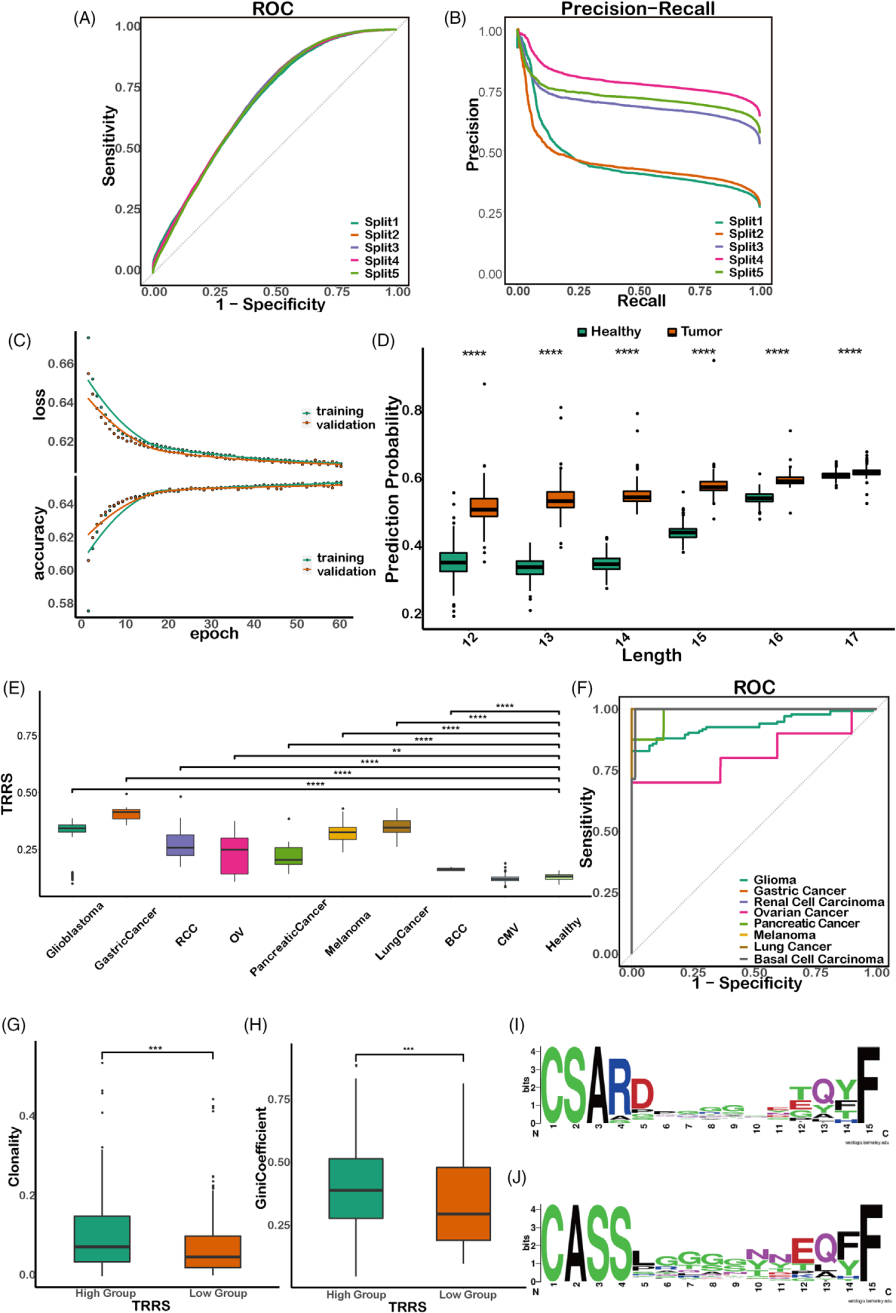
Development of binary model to predict tumour‐associated T cells (TATs) in the peripheral blood and using the T cell receptor repertoire (TCR) repertoire risk score (TRRS) to distinguish tumour patients from healthy individuals. (A–B) Model ROC and precision‐recall curve (PRC) by five‐fold cross validation. (C) The model loss/accuracy changes at different epochs. (D) The model prediction probability score for different CDR3 lengths among tumoural and non‐tumoural TCRs. (E) The TCR repertoire risk score (TRRS) in PBMC samples among tumour patients from different cancer types, healthy individuals from the Healthy69 cohort and human cytomegalovirus (HCMV) positive samples. (F) The ROC plot of TRRS in differentiating tumour patients from healthy donors at a given threshold of .66. (G–H) The indices of clonality and Gini coefficient between TRRS‐high group and TRRS‐low group. (I–J) The amino acid motif between TAT probability high group (top panel) and low group (bottom panel)

Then, to test whether the TAT prediction probability of our model can be used as a biomarker for differentiating tumour patients from healthy donors, we obtained seven independent datasets^55^, ^59^, ^60^, ^61^, ^62^, ^63^, ^64^ containing PBMC TCR sequencing data of patients with different cancer types (Figure S2A). The Healthy69 cohort was used as the negative controls set. We used a TRRS (Methods) to estimate the degree of TAT enrichment in the PBMC population of each sample. Briefly, we counted the number of TATs our model has predicted and then divided it by the number of healthy TCRs in PBMCs that overlapped with the Healthy351 pool.

At a threshold above .66, the TRRS can differentiate PBMC samples of normal individuals from those of patients with various cancer types effectively (Figure 2E,F), indicating the feasibility of using TRRS of TATs for non‐invasive tumour screening. We found that our result is robust by setting the threshold at different levels (Figure S3A–F). To provide evidence that the prediction of our model was not simply a generic active cell‐mediated immune response, we introduced an experimentally validated HCMV positive cohort with TCR‐seq data from their PBMC samples (Table S1). We calculated the TRRS for these individuals and compared them with that of the cancer patients. We show that the TRRS of HCMV positive cohort is lower than that of the cancer patients, indicating our model is able to distinguish individuals with cancer from those with infectious diseases. Based on the TRRS, we divided the samples in the independent validation cohort into high‐risk and low‐risk group separated at the 50% quantile. We found that the clonality and Gini coefficient in the high‐risk group were significantly higher (*p *< 1e^−3^) than those in the low‐risk group (Figure 2G,H), implying that the TCRs in the high‐risk group were associated with more clonal expansion and active immune functions. In addition, we analysed sequence motif enrichment in TCRs with the top 25% and bottom 25% probabilities of being a TAT (Figure 2I,J). We found that CDR3 sequences in the high probability group showed an enrichment of serine in the second position, while those in the low probability group tended to have an alanine in this position. Sending publicly available databases of virus/bacterial TCR sequences into the prediction model shows that CDR3 sequences among different lengths tended to have an alanine in the second position, which indicates that features specifically associated with the TCR of clonally expanded TATs are not enriched in virus/bacteria TCRs (Figure S3L). In summary, we used TCR sequences from TATs and healthy donors to build a binary predictive CNN model and designed a TRRS based on the model prediction for effective non‐invasive cancer screening with PBMCs.

Single‐cell‐RNA‐multiplexed‐TCR‐sequencing (scRNA‐TCR‐seq) technology makes it possible to not only trace the TCR clone sharing relationship between tumour and paired PBMCs samples, but also quantify the transcriptomics patterns at the single cell level.^43^, ^54^, ^65^, ^66^, ^67^, ^68^, ^69^ We performed a comprehensive literature review and obtained 14 high‐quality scRNA‐TCR‐seq datasets that met our criteria (Table S2). We found no significant difference (*p *> .05) in the CD4^+^/ CD8^+^ ratio between PBMCs from cancer patients and healthy donors (Figure 3A), implying that the relative ratio of CD4^+^ and CD8^+^ T cells remains unchanged after tumour initiation, in contrast to the ratio in patients with acute infection, which is usually lower than that in uninfected healthy samples.^70^, ^71^, ^72^ However, the proportion of clonal T cells (clone frequency > 2) was higher (*p *< 1e^−3^) in tumour patients than in healthy donors (Figure S4A), indicating a higher clonal expansion of T cells in cancer patients. Moreover, we found that the CD4^+^/ CD8^+^ ratio was significantly lower in TATs than in non‐clonal T cells (clone frequency = 1) in patient PBMCs (Figure 3B), suggesting that the expansion of CD8^+^ T cell is greater than that of the CD4^+^ cells upon tumour antigen stimulation.

**FIGURE 3:**
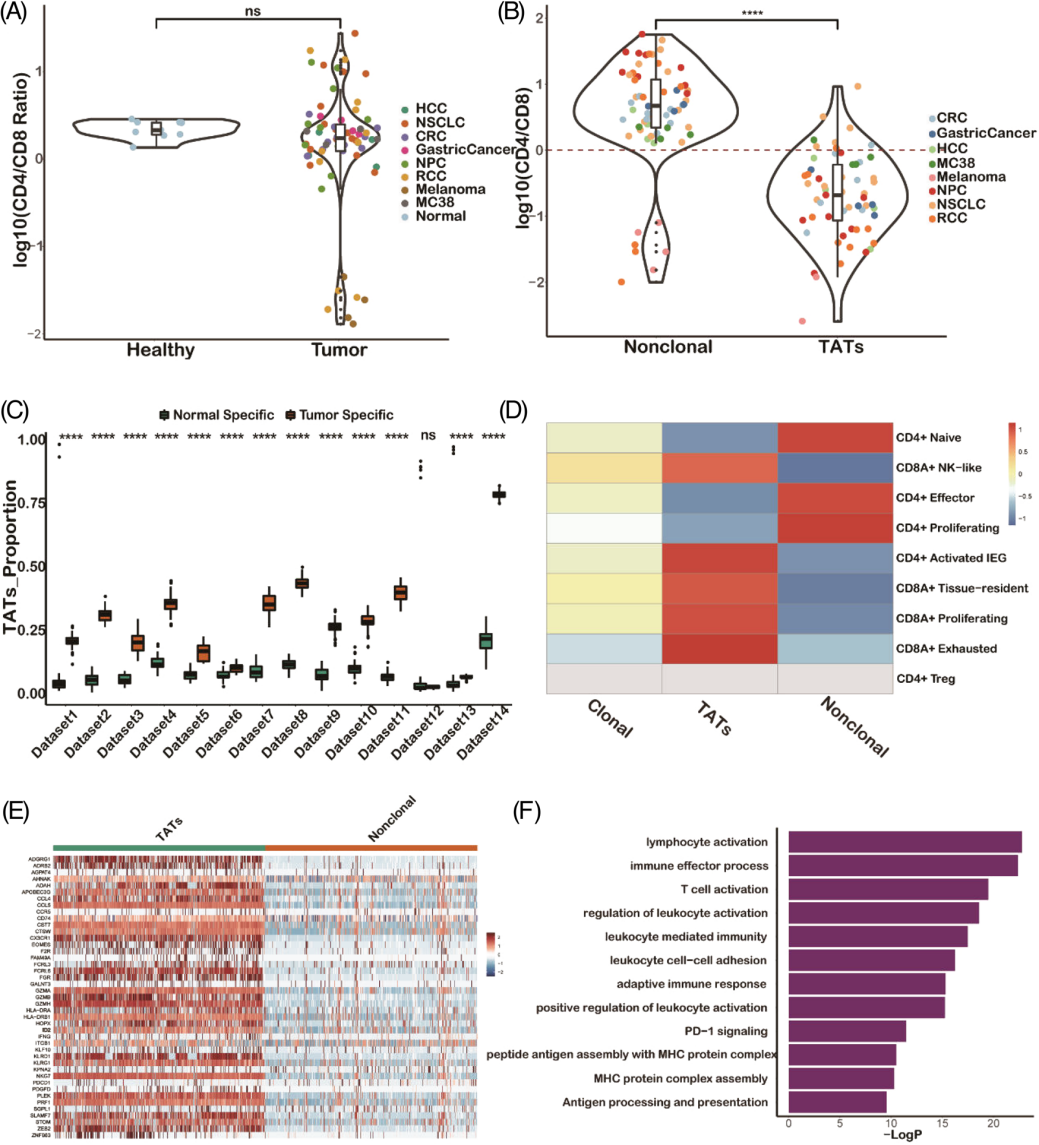
Transcriptional signature gene analysis using single cell data shows T cell activation is involved in the tumour‐associated T cells (TATs). (A) CD4^+^/ CD8^+^ T cell relative ratio in PBMCs between tumour and healthy samples calculated by single cell data. (B) CD4^+^/ CD8^+^ T cell relative ratio between TATs and non‐clonal T cells in PBMCs. (C) The proportion of TATs in tumour and healthy‐specific clusters (in which tumour patients or healthy donors derived T cells occupy more than 70%, see Methods) by TCR functional landscape estimation supervised with scRNA‐seq analysis (TESSA) clustering analysis. (D) T cell subtype distribution in different T cell receptor repertoire (TCR) compartments. (E) Heatmap shows differentially expressed genes between TATs and non‐clonal T cells. (F) Gene Ontology (GO) analysis of TAT differentially expressed genes

Next, to provide further evidence that TATs represent tumour‐specific T cells among PBMCs, we performed clustering analysis utilizing both single‐cell RNA and TCR information of T cells among tumour and healthy donor PBMCs by the TCR functional landscape estimation supervised with scRNA‐seq analysis (TESSA)^73^ algorithm. We found that tumour‐specific clusters had a higher proportion of TATs than normal specific clusters (*p *< 1e^−4^) in nearly all the 14 datasets (Figure 3C, Methods), indicating that TATs in the blood of patients with various types of cancer are tumour specific and dissimilar to T cells in the blood of healthy donors. To integrate datasets from various sources, we used a label transfer method^74^ by taking one clear cell renal cell carcinoma (ccRCC) dataset^75^ as the reference due to its detailed cell type annotation information. Then we projected the cells from other datasets onto the reference map to transfer the cell type annotation. We found that most T cells in the non‐clonal group were CD4^+^ naïve/proliferating/effector T cells, while most TATs were CD8^+^ NK‐like/effector T cells (Figure 3D). These results demonstrated that TATs are mostly activated CD8^+^ T cells and may exert cytotoxic functions upon tumour stimulation.

To further explore transcriptional signatures of TATs among PBMCs, we performed differential gene expression analysis between CD8^+^ TATs and non‐clonal T cells in each dataset. To prevent potential batch effects caused by using different data sources and guarantee a robust analysis, genes that were differentially expressed in more than 10 of the 14 datasets were selected to generate the TAT signature genes (Figure 3E). Enrichment analysis of TAT signature genes indeed implies a T lymphocytes activation and cytotoxicity function exemplified by pathways like antigen processing and presentation, PD‐1 signaling and immune effector process pathways, which are involved in anti‐tumour process (Figure 3F).

Similarly, T cell activation was enriched in non‐clonal group from patient PBMCs compared with that in T cells from healthy PBMCs (Figure S4B), indicating that non‐clonal T cells in tumour patients are generally more active than T cells in healthy donors. The reason might be two‐fold: (1) clonal T cells were labelled as non‐clonal due to sampling dropout in single‐cell experiments; (2) systematic immune response was induced upon tumour stimulation. Moreover, we found that the frequencies of TAT TCRs in tumour tissues were significantly correlated with those in PBMCs (Figure S4C), suggesting that the clone size of the TATs among PBMCs reflects the clone size in tumour sites to some extent.^48^

T cell metabolism is coupled with many immunological signals and facilitates the adaptation of T cells encountering pathogens and tumours.^76^ We mined Kyoto Encyclopedia of Genes and Genomes (KEGG) metabolism pathways and analysed metabolic pathway changes in TATs. We found that the glycosaminoglycan degradation pathway was significantly enriched (*p *< 1e^−3^) in TATs, while the purine metabolism pathway was significantly depleted (*p *< 1e^−3^) in TATs (Figure 4A). The glycosaminoglycan degradation pathway is mediated by enzymes produced by activated T cells, and it has been reported to be involved in the immune response and regulation of T cell homeostasis.^77^, ^78^ On the other hand, purine metabolism especially the adenosine synthesis axis serves as a common path for attenuating T cell activation and can mediate regulatory T cell to suppress immune activity^79^, ^80^, ^81^; therefore in the TATs compartment, this metabolic pathway is down‐regulated.

**FIGURE 4:**
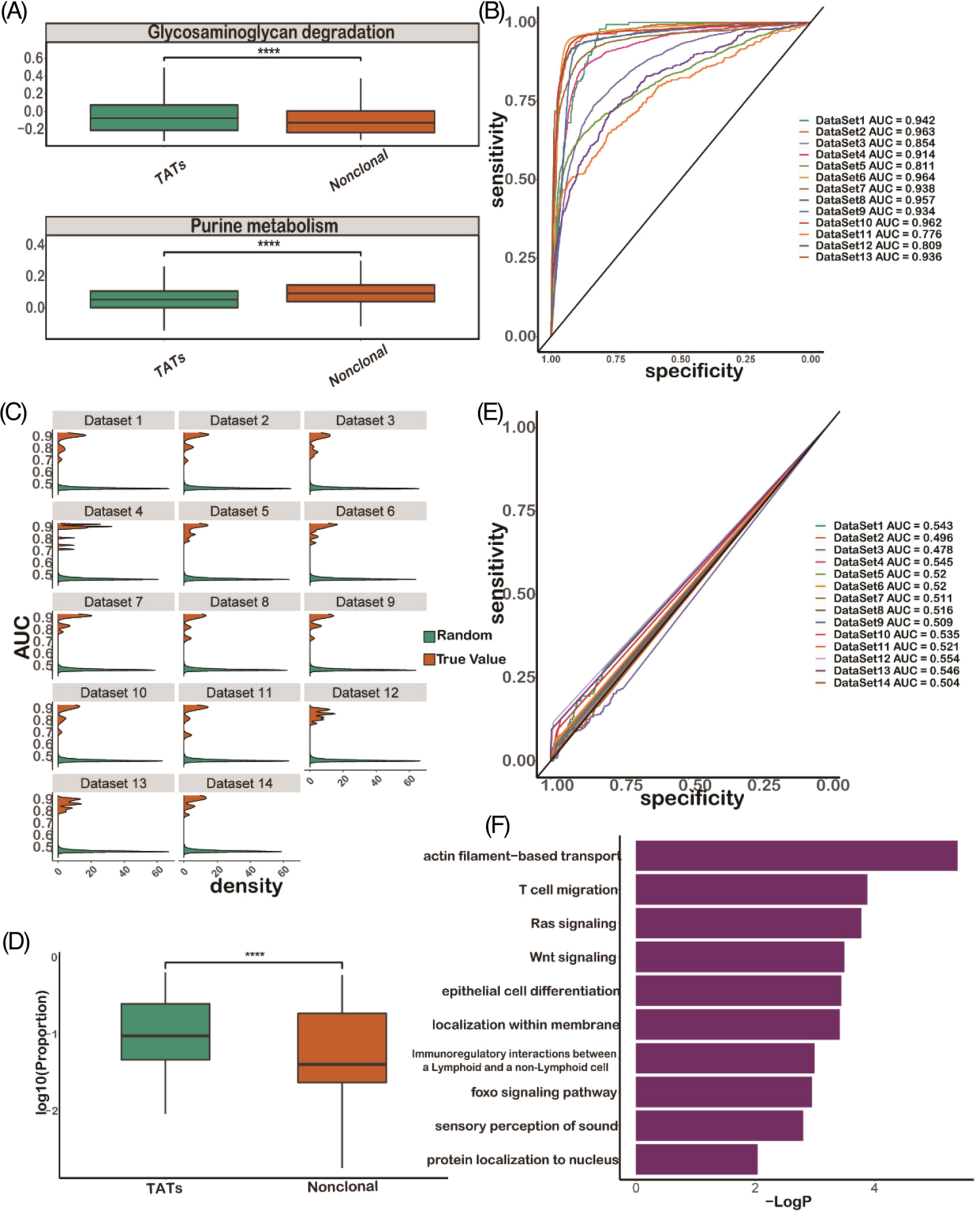
Transcriptional signatures are generalizable to predict the tumour‐associated T cells (TATs) from other single cell datasets. (A) Glycosaminoglycan degradation pathway is enriched in TATs, while the purine metabolism pathway is depleted in TATs. (B) ROC plot by using a colorectal cancer (CRC) dataset to train the prediction logistic regression model and testing the performance of model on the other datasets. (C) Permutation analysis by randomizing the true labels of TATs 1000 times and comparing the Area Under the Curve (AUC) distribution with the true prediction AUC values on each dataset. (D) PD‐1 expression between TATs and non‐clonal T cell group. (E) ROC plot using the expression of PD‐1 alone to differentiate TATs from non‐clonal T cells. (F) Differential expression analysis between TATs in PBMCs and *TILs_Shared* compartment in tumour‐infiltrated T lymphocytes (TILs)

Since the TAT data we collected were from samples manifesting different cancer types, with some obtained from different species, we wondered whether the transcriptional signatures of TATs in these datasets can be generalized to predict TATs in other datasets. We therefore performed a leave‐one‐dataset‐out cross‐validation experiment and used signatures of TATs explored in each dataset to build a logistic regression binary model. Then, we applied the model to independently test the TAT prediction performance on all the other datasets (Methods). The ROC demonstrated that the gene module can be satisfactorily generalized across different datasets (Figure 4B, Figure S4D–O) satisfactorily, and a permutation analysis demonstrated the robustness of our signatures (Figure 4C).

Our results suggest that using TCR sharing as molecular barcodes, we can characterize the transcriptional signatures of a group of tumour‐specific TATs. Previously, PD‐1^+^CD8^+^ T cells were believed to be tumour‐specific biomarkers in both TILs and PBMC population,^28^, ^29^ and we found that PD‐1 was indeed highly‐expressed in TATs (Figure 4D) compared with its expression in non‐clonal T cells (*p *< 1e^−4^). However, using the normalized expression of this gene alone was not sufficient to distinguish TATs from non‐clonal T cells (Figure 4E).

Because of the differences in TME and blood, TATs circulating in the periphery may acquire immunophenotypes that differ from those in TME, even when the TCR sequences are the same. Using the aforementioned procedure, we performed differential gene expression analysis between cells of TATs among PBMCs and T cells of *TILs_Shared* among TILs. We identified genes that were consistently differentially expressed in more than 10 datasets. We found that compared to T cells of *TILs_Shared* among tumour tissue, TATs among blood showed significantly higher levels of T cell migration and immunoregulatory interactions between lymphoid and non‐lymphoid cell (Figure 4F), further demonstrating that circulating TATs have the capacity to migrate across periphery, lymphoid and tumour tissues.

After identification of transcriptional signatures in TATs on the basis of scRNA‐TCR‐seq data, we sought to validate whether TAT signature genes can be applied to classify PBMC samples with bulk RNA‐seq. We collected three independent bulk RNA‐seq PBMCs datasets comprising 33 breast cancer PBMC samples, eight hepatocellular carcinoma (HCC) PBMC samples and 12 healthy PBMC samples. We named these datasets validation cohort 1 (Table S3). Principle component analysis showed that the tumour samples were transcriptionally separated from healthy samples (Figure 5A) in validation cohort 1, indicating salient differences between the peripheral blood of cancer patients and that of healthy individuals. We found that the expression pattern of TAT signature genes was distinct in tumour and healthy samples (Figure 5B), confirming that the signature module derived from the scRNA‐TCR‐seq data can also be used with bulk PBMCs RNA‐seq data to distinguish cancer patients from healthy individuals.

**FIGURE 5:**
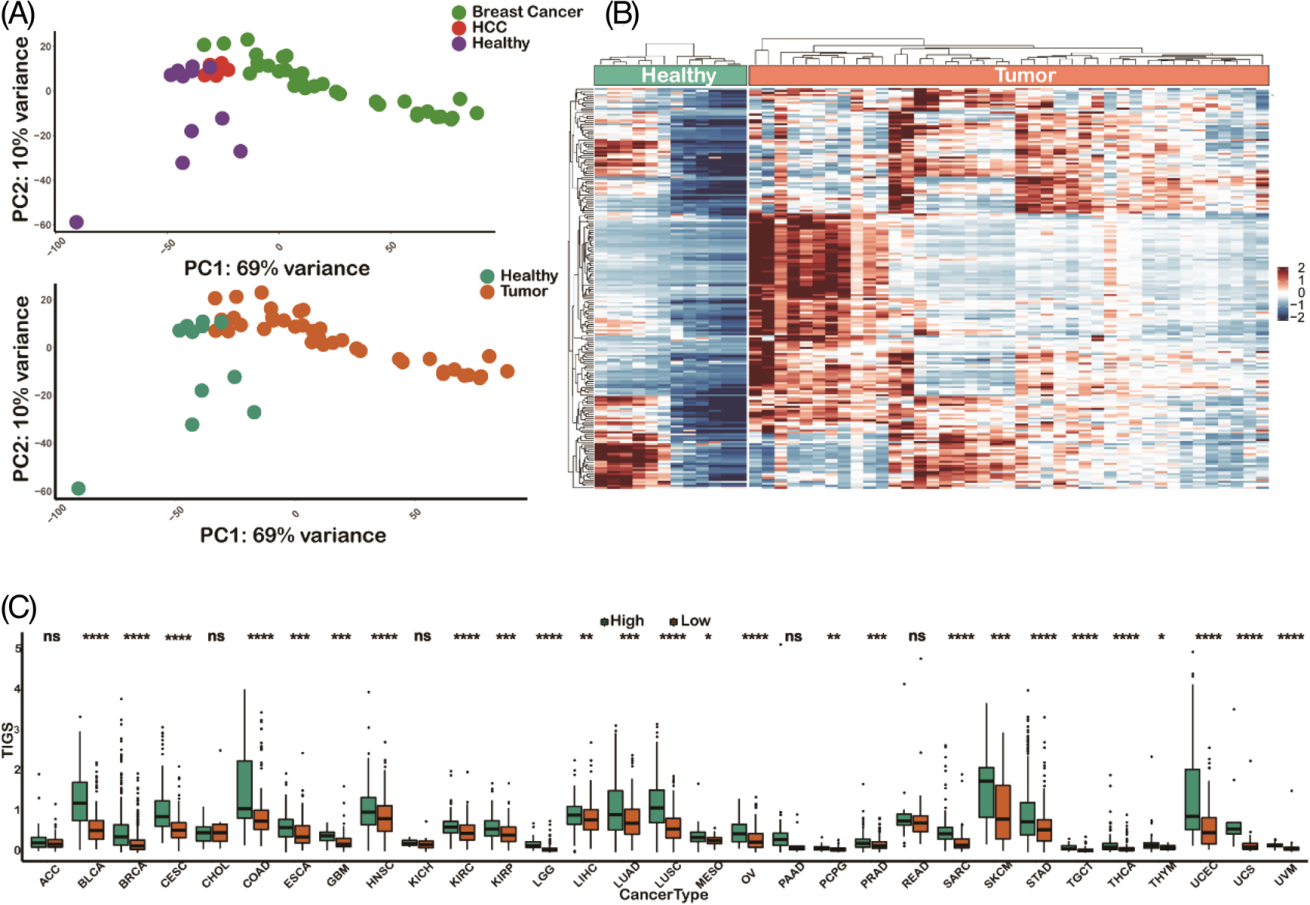
Transcriptional signatures acquired from single cell datasets can be used to distinguish tumour patients from healthy individuals of bulk RNA‐seq data. (A) Principle component analysis (PCA) plot of PBMC samples from tumour patients and healthy donors. (B) Heatmap of the expression patterns of tumour‐associated T cells (TATs) signature genes from tumour and healthy bulk RNA‐seq samples. (C) The tumour immunogenicity score (TIGS) in 31 cancer types from The Cancer Genome Atlas (TCGA) database between the TAT‐signature‐high group and the TAT‐signature‐low group

The tumour reactive immune response is usually triggered by immunogenic neoantigens expressed on tumour cells. Therefore, we sought to determine whether the tumour immunogenicity score (TIGS)^82^ is correlated with the expression of TAT signature genes. We utilized the mutation and transcriptional data of 31 cancer types in the The Cancer Genome Atlas (TCGA) database and defined the TIGS as the tumour mutation burden multiplied by the mean expression of a group of antigen‐presenting genes for each sample. We found that in nearly all the cancer types, the TIGS was significantly higher in the TAT‐signature‐high group than in the TAT‐signature‐low group (Figure 5C), providing evidence supporting our hypothesis that transcriptional activation of TATs is associated with the tumour mutation load.

Both the TCR repertoire and transcriptional signatures of TATs have the capacity to distinguish tumour patients from healthy samples. To determine whether the combination of these two types of features can be leveraged to achieve better performance for non‐invasive tumour screening, we performed bulk TCR and RNA sequencing of PBMCs samples from 11 tumour patients and six healthy donors (Table S4). Hereafter, we named this dataset as validation cohort 2. We merged the PBMC samples from validation cohort 1 and 2 to increase the sample number included in the integrated analysis. We found that the clonality and Gini coefficient of the PBMCs in cancer patients were significantly higher than those in healthy donors (Figure 6A,B), which is consistent with the results reported in the previous sections. Next, we predicted the relative proportion of immune cells in each sample by cell type devolution of the bulk RNA‐seq data using an R package, Immuno‐Oncology Biological Research (IOBR).^83^ We found that the fractions of T cells‐related components such as Tregs and T follicular helper cells were significantly different between tumour and healthy PBMCs samples (Figure 6C).

**FIGURE 6:**
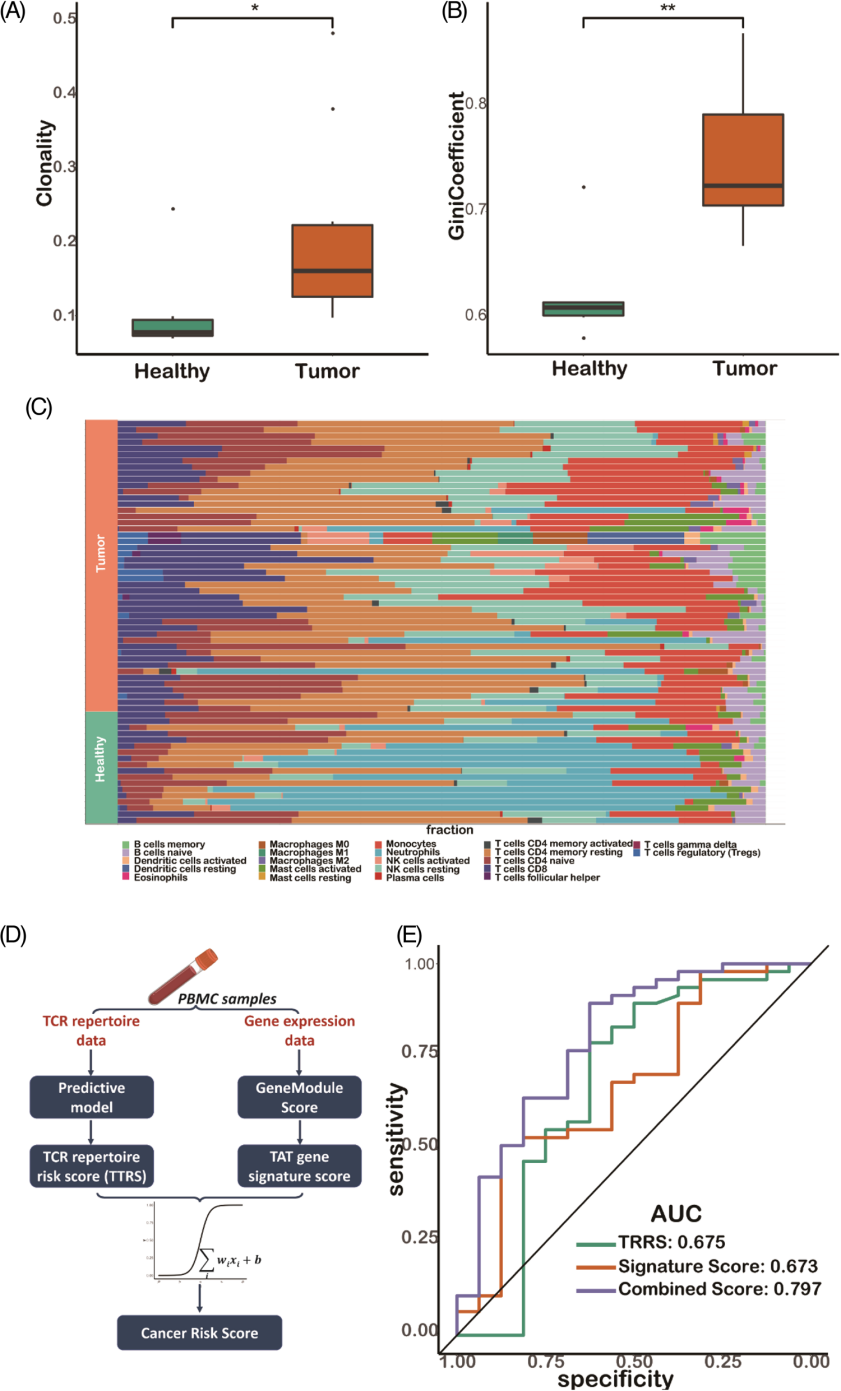
Combing both the T cell receptor repertoire (TCR) repertoire information and transcriptional signatures improves the capacity of non‐invasive tumour screening. (A–B) The indices of clonality and Gini coefficient among tumour and healthy samples in validation cohort 2 performed by bulk TCR sequencing. (C) Immune cell subtypes deconvoluted from bulk RNA‐seq data among PBMCs samples from tumour patients and healthy donors. (D) The schematic framework of combing TCR repertoire risk score (TRRS) and tumour‐associated T cells (TATs) signature gene score for tumour screening. (E) The ROC shows the model performance of using TCR repertoire risk score (TRRS) alone, using TAT signature score alone and using both of them for tumour screening

Next, by integrating these two types of features, we designed a novel tumour screening framework. We first used the TAT prediction model to generate the TRRS for each PBMC sample. Then, we calculated the TAT signature score using bulk RNA‐seq data obtained from the same sample. The two scores were then used to build a binary logistic regression model and define the final cancer risk score as the probability of the model prediction (Figure 6D). We found that either TRRS or TAT signature score has the capacity to distinguish blood samples of cancer patients from those of healthy individuals. Combining these two scores led to better predictive performance (Figure 6E), demonstrating the potential application of the two scores for use in non‐invasive tumour screening.

Understanding the tumour‐specific T lymphocyte response helps us explore immune signatures of tumourigenesis. In this study, we first separated the T cells into four different compartments based on the TCR‐sharing relationship and analysed the clone expansion differences between these compartments. We used a group of TATs in the PBMC population that shared TCRs with TILs and a group of healthy TCRs to build a binary model. We designed a TRRS, which is the number of predicted TATs among PBMCs divided by the number of healthy TCRs in the healthy TCR pool. We found that TRRS can serve as an indicator to distinguish tumour patients from healthy donors in a series of clinical cohorts with different cancer types. In addition, leveraging the scRNA‐TCR‐seq data of paired tumour tissues and PBMCs, we characterized the transcriptional signatures of TATs and found that T cell activity and cytotoxicity were increased in TATs. TAT signature genes mined from single cell data were also validated with bulk RNA‐seq PBMC data, showing high applicational prospect. Finally, combining the information from the TCR sequence and signature gene level of TATs, we designed an integrated framework for tumour screening and validated it with an independent clinical dataset. This framework considered tumour‐associated TCR repertoire and transcriptional patterns of TATs, providing insights into an alternative strategy of liquid biopsy on the basis of the immune cells.

Our method offers a new perspective for tumour screening harnessing the tumour‐associated immune response. Imaging‐based diagnostic assessments, including breast mammogram and low dose computed tomography scan screening methods, are used in limited cancer types.^84^, ^85^ False positive rate and overdiagnosis remain major concerns.^86^ Serum protein‐based markers such as α‐fetoprotein, prostate‐specific antigens, carcinoembryonic antigen, CA19–9 and CA125 have been investigated in clinical studies, but the specificity is not satisfactory for population level tumour screening.^1^, ^2^, ^3^ Liquid biopsy methods utilizing ctDNA usually need predefined biomarkers of tumour somatic mutations and are highly heterogeneous among and within cancer types. Mutations called from plasma cfDNA often come from white blood cells rather than tumour cells.^87^ Besides, tumour‐specific ctDNA and CTC are expected to be rare in the bloodstream, and capturing the tumour‐specific signals often needs to perform ultra‐deep next‐generation sequencing.^88^

Our method provides a feasibility of tumour screening from the view of tumour‐associated T cells that are antigen‐independent, shifting the diagnostic paradigm from tumour‐modality‐driven to immune‐response‐driven.^89^ Since immune responses are usually ahead of any measurable symptoms, this framework may have the potential to detect diseases at early stages. The melanoma cohort^60^ in our study consists of tumour samples of early stage (Stage I, Table S1), indicating the potential capability of our model for identifying early stage tumour patients. Besides, our framework can serve as a cheap, non‐redundant and complementary diagnostic paradigm for tumour screening since we use information of both DNA (for TCR repertoire quantification) and RNA (or protein, for immunophenotype quantification) level from peripheral blood. Last but not the least, the framework we provided is not intended to replace the current diagnostic paradigms, and we think it can serve as a complementary method with existing modalities.

There are limitations of our study, which should be considered. Our binary predictive model based on TCR sequences from different patients and cancer types does not currently address HLA restriction information. HLA restriction information is very important to the TCR repertoire distribution in patients since it is related to the pattern of the antigen peptides presented. We believe that, when it is accessible, this information can be incorporated into the model, and adding it will improve model performance. Leveraging deep learning architecture allows us to incorporate many types of features to predict TATs. However, we realize that transcriptional signatures are essential but not determinant factors for distinguishing tumours from healthy samples. The immune reaction induced by TSAs may be similar to that included by common pathogen infections. In our study, we have mined publicly available databases of virus/bacteria TCR sequences and removed all the T cell clones overlapped with the sequences in these databases before developing the model or performing differential gene expression analysis, alleviating this problem to a great extent. Notably, the starting material and library construction methods for TCR sequencing can profoundly influence the TCR repertoire detected. Using gDNA as the starting material for TCR sequencing leads to more stable results and allows for better quantification of each single TCR clone, while employing RNA potentially provides information on expression levels. Therefore, consolidating TCR sequencing data from different starting materials and library construction warrants further efforts.

## CONFLICT OF INTEREST

The authors declare no potential conflict of interest.

## Supporting information

Supporting Information

Supporting Information
