# Supplementary material for: TCR repertoire and transcriptional signatures of circulating tumour‐associated T cells facilitate effective non‐invasive cancer detection
Source: Clin Transl Med. 2022 Sep 22;12(9):e853. doi: 10.1002/ctm2.853 (PMC9494610; doi:10.1002/ctm2.853)
Supplement: Supplementary file 2 — Supporting Information [file CTM2-12-e853-s002.docx]

**Title:**

TCR repertoire and transcriptional signatures of circulating Tumor-Associated T cells facilitate effective noninvasive cancer detection.

**Letter-to-Editor with previous submission number CTM2-2022-01-0044.**

**Authors and affiliations:**

Fansen Ji ^a, c, *^, Lin Chen ^b, c, *^, Zhizhuo Chen ^d^, Bin Luo ^e^, Yongwang Wang ^f^, Xun Lan^c, g^.

**^a^** Tsinghua-Peking Center for Life Sciences, MOE key laboratory of Tsinghua University, Beijing, China. jfs18@mails.tsinghua.edu.cn.

**^b^** General Surgery Department, Beijing Tsinghua Changgung Hospital, School of Clinical Medicine, Tsinghua University, Beijing China. cla03110@btch.edu.cn

**^c^** School of Medicine, Tsinghua University, Beijing, China.

**^d^** School of Life Science, Tsinghua University, Beijing, China. czz19@mails.tsinghua.edu.cn.

**^e^** General Surgery Department, Beijing Tsinghua Changgung Hospital, School of Clinical Medicine, Tsinghua University, Beijing China, corresponding author, luobin@mail.tsinghua.edu.cn.

**^f^** Affiliated Hospital of Guilin Medical University, Department of Anesthesiology, Guilin China, corresponding author, wangyongwang81@126.com.

**^g^** Tsinghua-Peking Center for Life Sciences, MOE key laboratory of Tsinghua University, Beijing, China, main corresponding author. xlan@tsinghua.edu.cn.

**^*^** These authors contributed equally: Fansen Ji, Lin Chen.

**Running Title:**

Tumor-Associated T cells for tumor screening.

**Acknowledgements**

We would like to thank the ImmuneAccess, TCGA and GEO databases for the availability of the data. This work was supported by the Tsinghua University-Peking University Jointed Center for Life Science [Grant No. 61020100119 to X.L.]; National Thousand Young Talents Program of China [Grant No. 042021011 to X.L.]. We thank American Journal Experts (AJE) for English language editing.

**Corresponding Authors:**

**Xun Lan**, School of Medicine, Medical Science Building, Tsinghua University, Haidian District, Beijing 100084, China.

Phone: 86-10-62770517;

Fax: 86-10-62791875;

E-mail: xlan@tsinghua.edu.cn
**Bin Luo**, Beijing Tsinghua Changgung Hospital, Room 431, Building 3, 168 Litang Road, Changping District, Beijing, 102218, China.

Beijing 100084, China.

Phone: 86-10-56118937;

Fax: 86-10-56118550.;

E-mail: luobin@mail.tsinghua.edu.cn

**Yongwang Wang**, Affiliated Hospital of Guilin Medical University, Department of Anesthesiology, No.15 Lequn Road, Xiufeng District, Guilin, 541001, China.

Phone: 86-131-0205-4966;

Fax: 0773-2833802;

E-mail: wangyongwang81@126.com

**Conflict of interest statement:** The authors declare no potential conflicts of interest.

**Materials and Methods**

***Data curation and preprocessing.***

Bulk CDR3 beta chain sequencing data of both tumor tissues and PBMCs from different cancer types was collected from [immuneACCESS database](https://clients.adaptivebiotech.com/immuneaccess). Healthy donor PBMCs TCR data was collected from a large-scale cohort [1]. Healthy donor data of HCMV negative status consists of two cohorts according to the original paper and we named these two batches of dataset as Healthy351 and Healthy69 based on the sample number. Virus associated TCR data was collected from public databases [2–5]. The detailed data summary can be found in **Table S1**. scRNA-TCR-seq data from both tumor and PBMCs samples for the same cancer patient was collected from a series of public papers and summarized in **Table S2**. Public PBMCs bulk RNA-seq data was collected from different papers and detailed information can be found in **Table S3**. Complete productive CDR3 beta chains defined as the last cysteine (C) in the variable gene to the first Phenylalanine in the FGXG motif in the joining gene. Non-productive TCR sequences (due to frame shift or stop codon within the sequences) were removed and TCRs that were detected in the curated virus associated databases were also removed. Bulk RNA-seq data was preprocessed using the STAR [6] software and DEseq2 [7] package for counts quantification while using TRUST4 [8] for TCR sequence assembly. Seurat [9] *AddModuleScore* function was used to calculate the gene module score for each sample based on the signatures derived from scRNA-TCR-seq data.

***TCR sequence data analysis.***

TCR clonotypes were defined as a group of TCRs that have the same CDR3 beta chain amino acids. For bulk TCR-seq data, clonality and Gini coefficient were calculated using the LymphoSeq R package (https://github.com/davidcoffey/LymphoSeq). For scTCR-seq data, the clonal proportion is calculated by dividing the number of clonal TCRs (clonal frequency > 2) by the total number of TCRs in the corresponding compartment. CDR3 motif analysis was plotted using the website (https://weblogo.berkeley.edu/logo.cgi). CDR3 V gene usage data was normalized the sequence reads depth for each patient and the reads count for each T cell compartment and plotted using tidyHeatmap R package (https://github.com/stemangiola/tidyHeatmap).

***TCR beta chain featurization and model development using CNN.***

Paired PBMCs and TILs TCR-seq data for the same patient was firstly intersected and then reserved unique clonotypes for further analysis. We excluded virus associated sequences or TCR clones that were also emerged in Healthy351. These unique CDR3 beta chain clonotypes were labeled as positive or tumor associated TCRs. Besides, the same number of CDR3 beta chain sequences were sampled from Healthy351 and were then labeled as negative or healthy TCRs. Since the length of CDR3 beta chain sequences in our project is between 12 to 17, we modeled this problem by right padding to set the length of each CDR3 beta chain sequence as 17. Then, we used one-hot encoding method to transform each amino acid of CDR3 beta chain sequence into numeric space as to provide a discrete representation of amino acids.

The resulting input image is 17*20 for each CDR3 sequence and was then sent into the first convolutional layer with 8 17-by-2 filters. A rectified linear unit (ReLU) activation and a maximum pooling layer with pooling size of 1-by-2 and stride 2 were subsequently applied. The second convolutional layer with 16 1-by-2 filters followed by an additional same ReLU activation and maximum pooling layer were also applied. A dense layer with 10 units and ReLU activation layer was added on top of the convolutional layers while a SoftMax functions was applied to the final output layer in order to export probabilities related to class labels, which were TATs or Healthy. We used 5-fold cross validation based on the tumor patients rather than TCR sequences and randomly selected 50% for training and 25% for test/validation for each split. Adam Optimizer was used to minimize the binary cross entropy loss of CNN model until convergence criteria were met.

***Validating the influence of HLA haplotype, sex and age on the model development.***

It is conceivable that HLA haplotypes may influence the TCR repertoire distribution because they directly impact the antigen presentation. At present, the HLA allele information for each sample in our research is very limited. We have mined the corresponding papers and collected the related clinical information especially HLA type if it is available (Table S1). Here, we chose HLA-A*02 and HLA-B*15 since these two alleles not only constitute a relatively high fraction of the population[10, 11] but also appeared in the collected datasets. Then, we used TAT TCRs from individuals without HLA allele information to train the CNN model and test the performance of model on TCRs from the HLA-allele-carriers.

***Definition of a TCR repertoire risk score (TRRS).***

Since most of TCR sequences in the periphery represent naïve T cells and they are not tumor specific, deciding whether the patient is at high risk of cancer is somewhat tracking tumor specific signals over a pool of irrelevant TCRs and needs to aggregate the whole TCR repertoire. Here, we designed a TCR repertoire risk score to reflect the enrichment of TATs for each individual in PBMCs. We firstly used our binary predictive model to get a probability for each TCR in the PBMCs for a certain individual, then at a given cutoff we counted the number of TATs in the whole TCR repertoire (denoted as ***x***). Next, we counted the number of TCRs in PBMCs from this individual that have also been observed in Healthy351 cohort (denoted ***y***) to reflect the healthy associated immune response. The final TCR repertoire risk score is calculated by ***x*** dividing by ***y***. We used the number of truly healthy TCRs that have been detected in a large healthy individual cohort to calibrate the TATs that our model has predicted. If this proportion is very high, we believed the sample is more likely to have tumor. After model construction, we used independent bulk TCR-seq data from tumor PBMCs samples to evaluate the model performance. Patients in the independent validation cohort were divided into high-risk group and low-risk group according to the 50% TRRS quantile.

***Clustering analysis of PBMCs T cells from scRNA-TCR-seq data.***

We adopted a recent public TESSA clustering algorithm [12] by combining both RNA and TCR information of each single cell. After clustering analysis, clusters containing more than 70% T cells that are from tumor PBMCs samples are defined as tumor-specific clusters while clusters containing more than 70% T cells that are from normal PBMCs samples are defined as normal-specific clusters. We believe that healthy specific T cell clusters can be regarded as a baseline of immune response without tumor stimulation while tumor specific clusters are supposed to more likely enrich tumor reactive T cells. Then, we inspected T cells from both the tumor specific and normal specific clusters to check their cell identities (TATs or nonclonal) and calculated the relative ratio of these two compartments.

***Identification of T cell subtype from scRNA-TCR-seq data.***

Since we have multiple scRNA-TCR-seq datasets, merging all of them together for T cell type identification will introduce huge batch effect. Here, we used a reference-query label transfer method developed by Seurat [9]. Firstly, we selected one dataset of ccRCC [13] that have relatively concise and detailed cell type annotation information and regarded it as a reference map. Then, we made every other dataset as a query dataset to acquire the predicted cell type labels. Next, we normalized the relative proportion of each cell type in each compartment and finally merged all the proportion together to get a mean proportion. Differential gene expression analysis was done by using FindMarkers function of Seurat [9].

***Prediction of TATs using scRNA-TCR-seq data.***

In order to test whether the differential expressed genes between TATs and nonclonal can be generalized on the other datasets, we selected each of the 14 scRNA-TCR-seq datasets as training data and did gene differential expression analysis on this training dataset between TATs and nonclonal. Then we used the gene signatures explored from this dataset to build a binary logistic regression model to classify the T cell identities (TATs or nonclonal). It should be noted that all T cells were aggregated by TCR clones. Next, we applied the trained model independently onto the other 13 datasets to test whether the genes found in one dataset can be generalized on other datasets and we plotted the ROC curve to present the model performance. In order to test the significance or robustness of our model, we did permutation analysis for each test dataset 1000 times by randomizing the true label of T cell clones.

***Experimental framework for noninvasive tumor screening***

PBMC sample acquired from each individual is firstly divided into two parts, and one is sent to perform bulk TCR-sequencing while the other is sent to perform bulk RNA-sequencing. Then we used the TAT prediction model to calculate the TCR repertoire risk score and calculated the TAT signature score using bulk RNA-seq data obtained from the same sample. TCR sequencing data can also be assembled using TRUST4 [8]. The two scores for each sample can serve as two independent features to build a binary logistic regression model. The final cancer risk score is defined as the probability of the model prediction. Once a new PBMC sample is acquired, we can use the whole framework described above to estimate the risk of having cancer.

***Clinical sample processing and sequencing.***

PBMCs samples from cancer samples were collected into K_2_EDTA tubes at the time of surgeries and haven’t received any neoadjuvant treatments before. PBMCs were extracted using Ficoll-Paque PLUS (GE Healthcare). Then, total RNA was isolated and purified using TRIzol reagent (Invitrogen, Carlsbad, CA, USA) following the manufacturer's procedure. 2×150bp read pairs were sequenced (PE150) on an illumina Novaseq™ 6000 (LC-Bio Technology CO., Ltd., Hangzhou, China) following the vendor's recommended protocol. DNA samples PBMCs from the same patient were analyzed by high-throughput sequencing of TCR beta chain using the ImmuHub TCR profiling system at a deep level (ImmuQuad Biotech, Hangzhou, China).

***Immune cell deconvolution and integrated model development.***

We collected three independent bulk RNA-seq PBMCs datasets comprising 33 breast cancer PBMC samples, 8 hepatocellular carcinoma (HCC) PBMC samples and 12 healthy PBMC samples (**Table S3**). We named this dataset as validation cohort 1. We have also performed bulk TCR and RNA sequencing of PBMCs samples from 11 tumor patients and 6 healthy donors (**Table S4**). We predicted the relative proportion of immune cells by cell type deconvolution [14] of the bulk RNA-seq data combining both validation cohort 1 and validation cohort 2. We had also merged these two cohorts to develop an integrated noninvasive model. We used TRUST4 algorithm [8] to assemble TCR sequences of bulk RNA-seq data from validation cohort 1. We next used the TAT prediction model to generate the TCR repertoire risk score for each PBMC sample. Then, we calculated the TAT signature score using bulk RNA-seq data obtained from the same sample. The two scores were then used to build a binary logistic regression model and define the final cancer risk score as the probability of the model prediction.

**Acknowledgements**

We would like to thank the ImmuneAccess, TCGA and GEO databases for the availability of the data. This work was supported by the Tsinghua University-Peking University Jointed Center for Life Science [Grant No. 61020100119 to X.L.]; National Thousand Young Talents Program of China [Grant No. 042021011 to X.L.]. We thank American Journal Experts (AJE) for English language editing.

**Declaration of competing interest**

The authors declare that they have no known competing financial interests or personal relationships that could have appeared to influence the work reported in this paper.

**Ethics Approval and Consent to Participate**

In this work, PBMCs samples from cancer patients and healthy donors were collected from Beijing Tsinghua Changgung Hospital and informed consent was obtained from donors. This study was approved by the Ethical Committee of Beijing Tsinghua Changgung Hospital (No. 21405-6-01) and compiled with all legal ethical regulations. All samples provided written informed consent for the collection of blood samples for research and TCR/RNA profiling.

**Data Availability Statement**

The Sequencing raw data of bulk RNA-seq and TCR-seq for validation is available generated in this study are publicly available at GSA (Genome Sequence Archive in BIG Data Center, Beijing Institute of Genomics, Chinese Academy of Sciences). The accession number is HRA001616. The other TCR-seq data and scRNA-seq-TCR-seq data is public available and have been summarized in the **Table S1** and **Table S2**. Custom code to repeat the data analysis is available from corresponding authors upon reasonable request.

**Author contributions**

FSJ designed this study. FSJ and LC drafted the manuscript and conducted the statistical analysis. ZZC revised this manuscript. All authors read and approved the final manuscript.

**Reference**

1. Emerson RO, DeWitt WS, Vignali M, et al (2017) Immunosequencing identifies signatures of cytomegalovirus exposure history and HLA-mediated effects on the T cell repertoire. Nat Genet 49:659–665. https://doi.org/10.1038/ng.3822

2. Bagaev DV, Vroomans RMA, Samir J, et al (2020) VDJdb in 2019: database extension, new analysis infrastructure and a T-cell receptor motif compendium. Nucleic Acids Res 48:D1057–D1062. https://doi.org/10.1093/nar/gkz874

3. Dhanda SK, Mahajan S, Paul S, et al (2019) IEDB-AR: immune epitope database—analysis resource in 2019. Nucleic Acids Res 47:W502–W506. https://doi.org/10.1093/nar/gkz452

4. Tickotsky N, Sagiv T, Prilusky J, et al (2017) McPAS-TCR: a manually curated catalogue of pathology-associated T cell receptor sequences. Bioinforma Oxf Engl 33:2924–2929. https://doi.org/10.1093/bioinformatics/btx286

5. Zhang W, Wang L, Liu K, et al (2020) PIRD: Pan Immune Repertoire Database. Bioinforma Oxf Engl 36:897–903. https://doi.org/10.1093/bioinformatics/btz614

6. Dobin A, Davis CA, Schlesinger F, et al (2013) STAR: ultrafast universal RNA-seq aligner. Bioinforma Oxf Engl 29:15–21. https://doi.org/10.1093/bioinformatics/bts635

7. Moderated estimation of fold change and dispersion for RNA-seq data with DESeq2 | Genome Biology | Full Text. https://genomebiology.biomedcentral.com/articles/10.1186/s13059-014-0550-8. Accessed 13 Dec 2021

8. Song L, Cohen D, Ouyang Z, et al (2021) TRUST4: immune repertoire reconstruction from bulk and single-cell RNA-seq data. Nat Methods 18:627–630. https://doi.org/10.1038/s41592-021-01142-2

9. Hao Y, Hao S, Andersen-Nissen E, et al (2021) Integrated analysis of multimodal single-cell data. Cell 184:3573-3587.e29. https://doi.org/10.1016/j.cell.2021.04.048

10. Puangpetch A, Koomdee N, Chamnanphol M, et al (2015) HLA-B allele and haplotype diversity among Thai patients identified by PCR-SSOP: evidence for high risk of drug-induced hypersensitivity. Front Genet 5:

11. Esmaeili A, Rabe SZT, Mahmoudi M, Rastin M (2017) Frequencies of HLA-A, B and DRB1 alleles in a large normal population living in the city of Mashhad, Northeastern Iran. Iran J Basic Med Sci 20:940–943. https://doi.org/10.22038/IJBMS.2017.9117

12. Zhang Z, Xiong D, Wang X, et al (2021) Mapping the functional landscape of T cell receptor repertoires by single-T cell transcriptomics. Nat Methods 18:92–99. https://doi.org/10.1038/s41592-020-01020-3

13. Krishna C, DiNatale RG, Kuo F, et al (2021) Single-cell sequencing links multiregional immune landscapes and tissue-resident T cells in ccRCC to tumor topology and therapy efficacy. Cancer Cell. https://doi.org/10.1016/j.ccell.2021.03.007

14. Zeng D, Ye Z, Shen R, et al (2021) IOBR: Multi-Omics Immuno-Oncology Biological Research to Decode Tumor Microenvironment and Signatures. Front Immunol 12:2547. https://doi.org/10.3389/fimmu.2021.687975


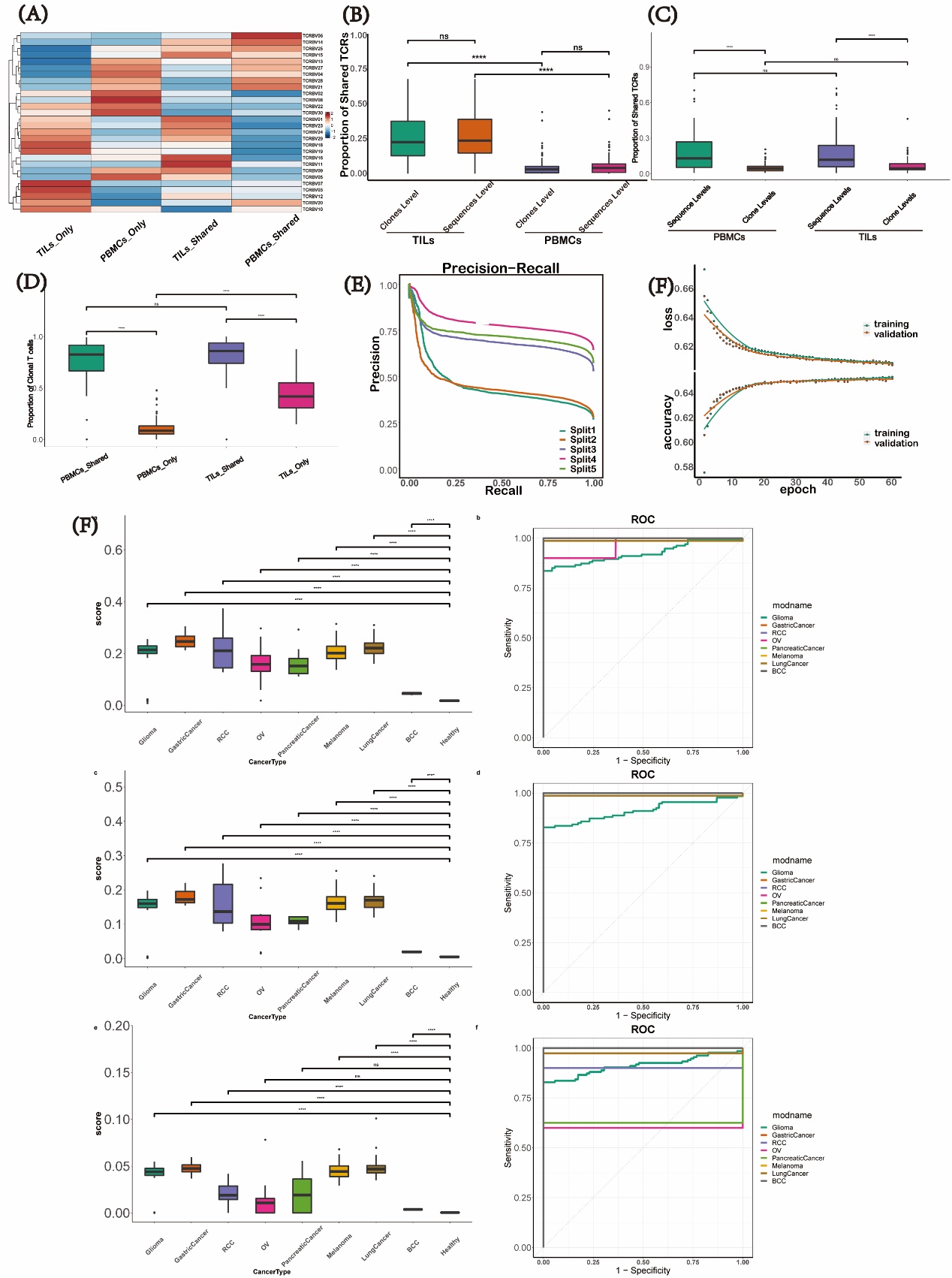


**Figure S1 T cell receptor (TCR) repertoire can serve as a noninvasive tumor biomarker to distinguish tumor patients from healthy individuals. A**: V gene usage of the CDR3 beta chains in different TCR compartments. **B:** Proportion of shared TCRs among different tissue types (TILs or PBMCs). The result is consistent both at sequence level or clone level. **C:** Proportion of shared TCRs in different tissue types (TILs or PBMCs) using scTCR-seq data. The proportion of shared TCRs is calculated at both TCR sequence level and clone level. **D:** The proportion of clonal TCRs (Clone frequency >2) in different TCR compartments using scTCR-seq data. E: Precision-Recall curve (PRC) by 5-fold cross validation. **E:** The model loss/accuracy changes reached to a plateau after about 60 epochs. **F:** The TCR repertoire risk score (TRRS) distribution and ROC performance under different model threshold (0.75, 0.85, 0.95).


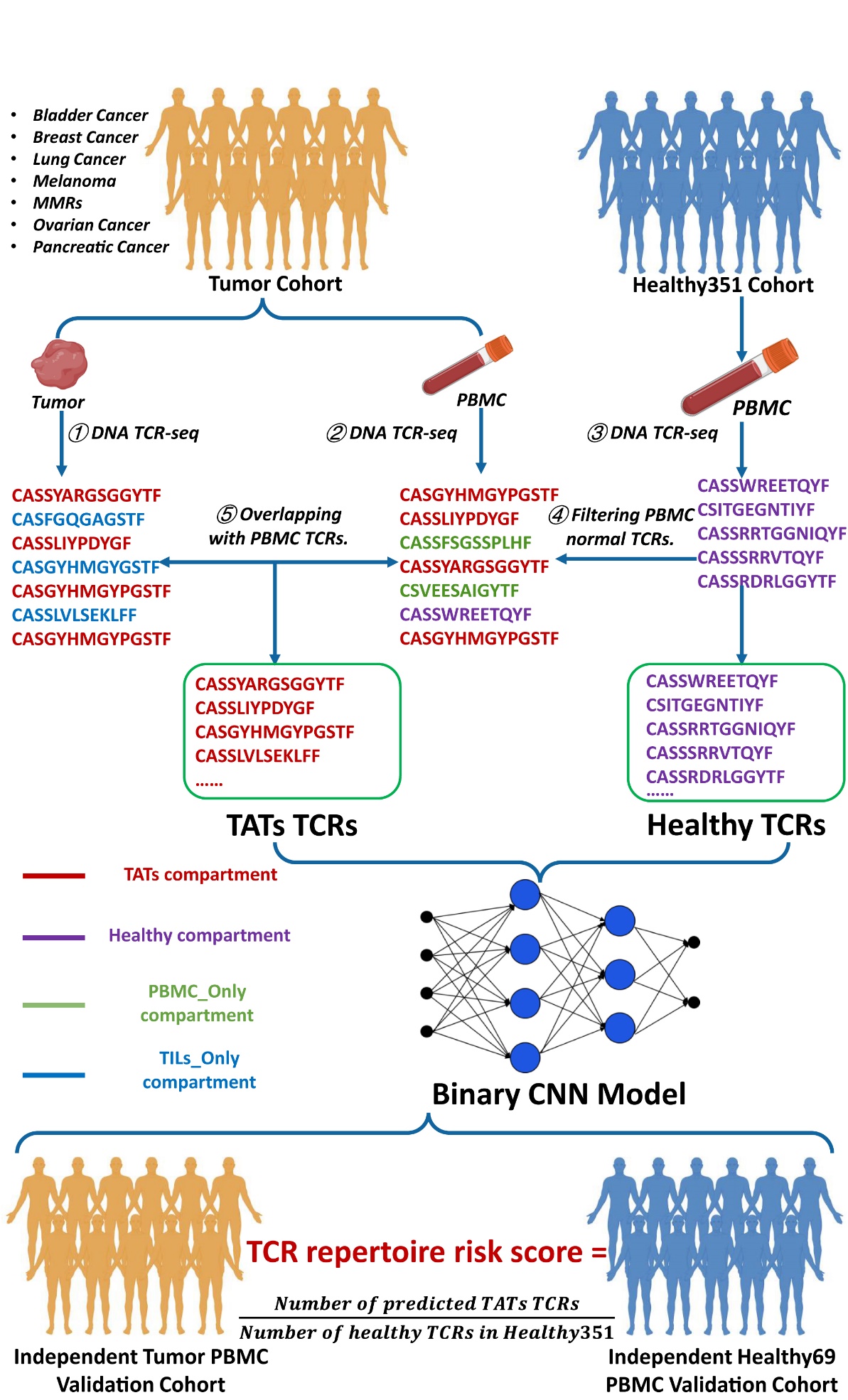


**Figure S2.** Schematic workflow of the CNN model development to predict TATs and the TCR repertoire risk score (TRRS) calculation.


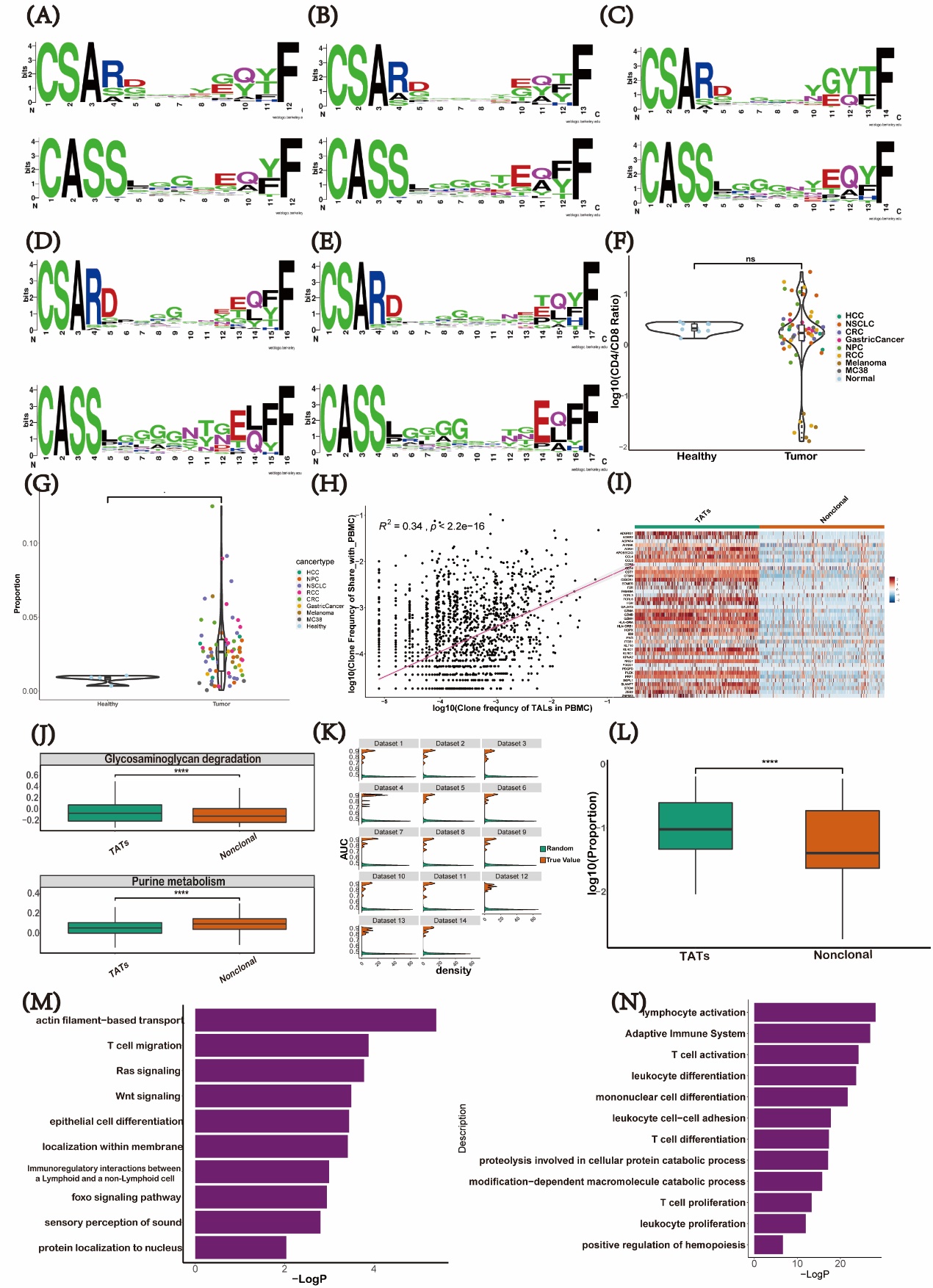


**Figure S3.** **A-E**: The TCR logo motif under different CDR3 length in high TAT probability group and low TAT probability group. **F:** CD4^+^/ CD8^+^ T cell relative ratio in PBMCs between tumor and healthy samples calculated by single cell data. **G:** The proportion of clonal T cells (clone frequency>2) in tumor and healthy PBMCs. **H:** The clone frequency of each clonotype in tumor and PBMCs tissues shows a positive correlation. **I:** Heatmap shows differentially expressed genes between TATs and nonclonal T cells. **J:** Glycosaminoglycan degradation pathway is enriched in TATs while the purine metabolism pathway is depleted in TATs. **K:** Permutation analysis shows logistic regression model using signatures of TATs in each dataset can be significantly generalized to predict TATs in other datasets. **L:** PD-1 expression is up-regulated in TATs compared with that in nonclonal T cell group. **M:** Differential gene expression analysis between TATs in PBMCs and TILs_Shared compartment in TILs shows that T cell migration pathways as well as interactions between lymphoid and non-lymphoid tissues are enriched in TATs. **N:** Differential gene expression analysis between nonclonal T cells in tumor PBMCs and healthy T cells shows relatively high T cell activation in nonclonal T cells in tumor PBMCs.


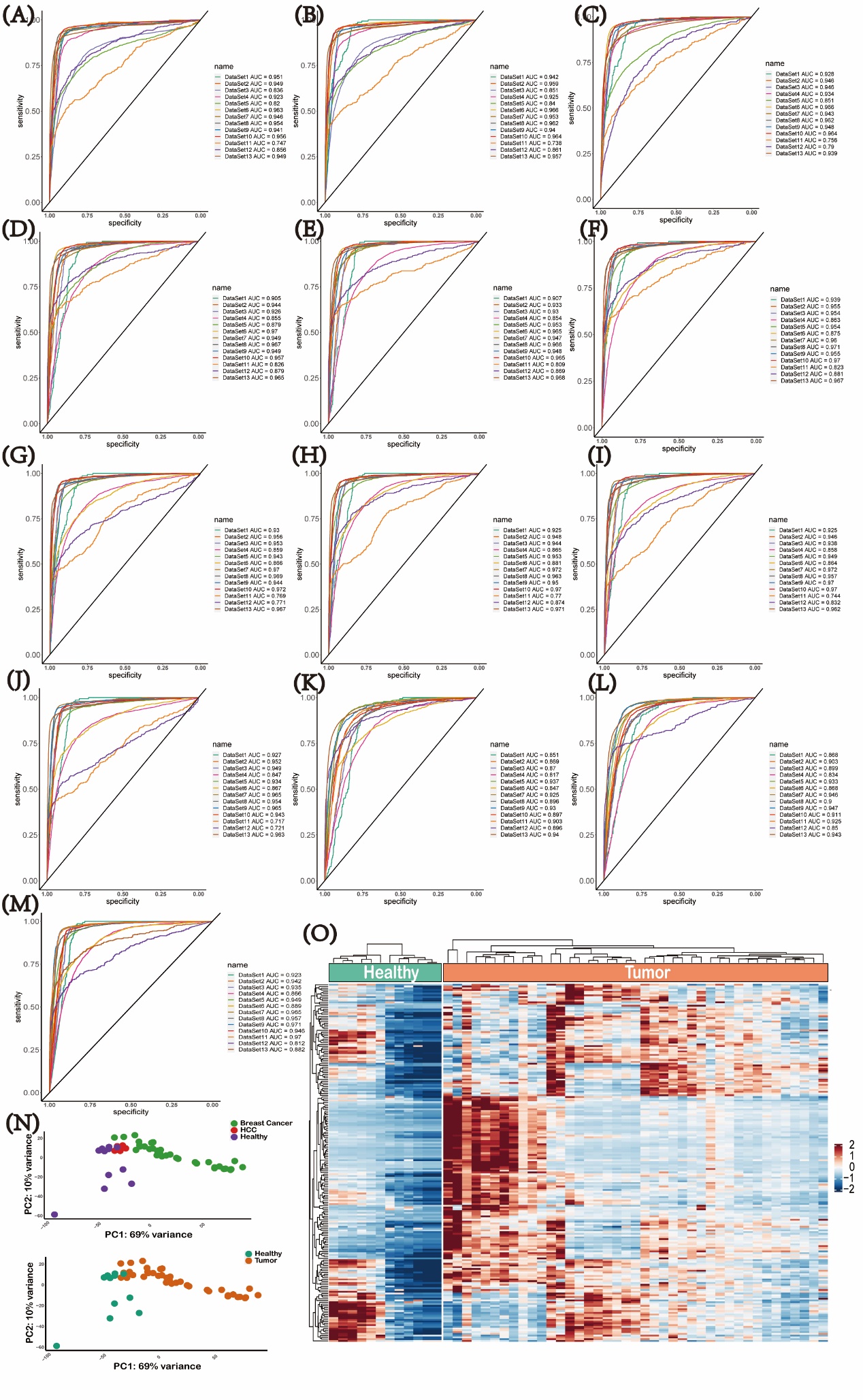


**Figure S4. A-M:** The model performance by using signatures of one dataset to predict TATs in the other datasets. **N:** PCA plot shows that the tumor samples were transcriptionally separated from healthy samples. O: Heatmap shows the expression pattern of TAT signature genes was distinct in tumor and healthy bulk RNA-seq samples.
